# Supplementary material for: Discovery and ramifications of incidental Magnéli phase generation and release from industrial coal-burning
Source: Nat Commun. 2017 Aug 8;8:194. doi: 10.1038/s41467-017-00276-2 (PMC5548795; doi:10.1038/s41467-017-00276-2)
Supplement: Supplementary file 1 — Supplementary Information [file 41467_2017_276_MOESM1_ESM.pdf]

File name: Supplementary Information
Description: Supplementary Figures and Supplementary Tables

File name: Peer Review File
Description:

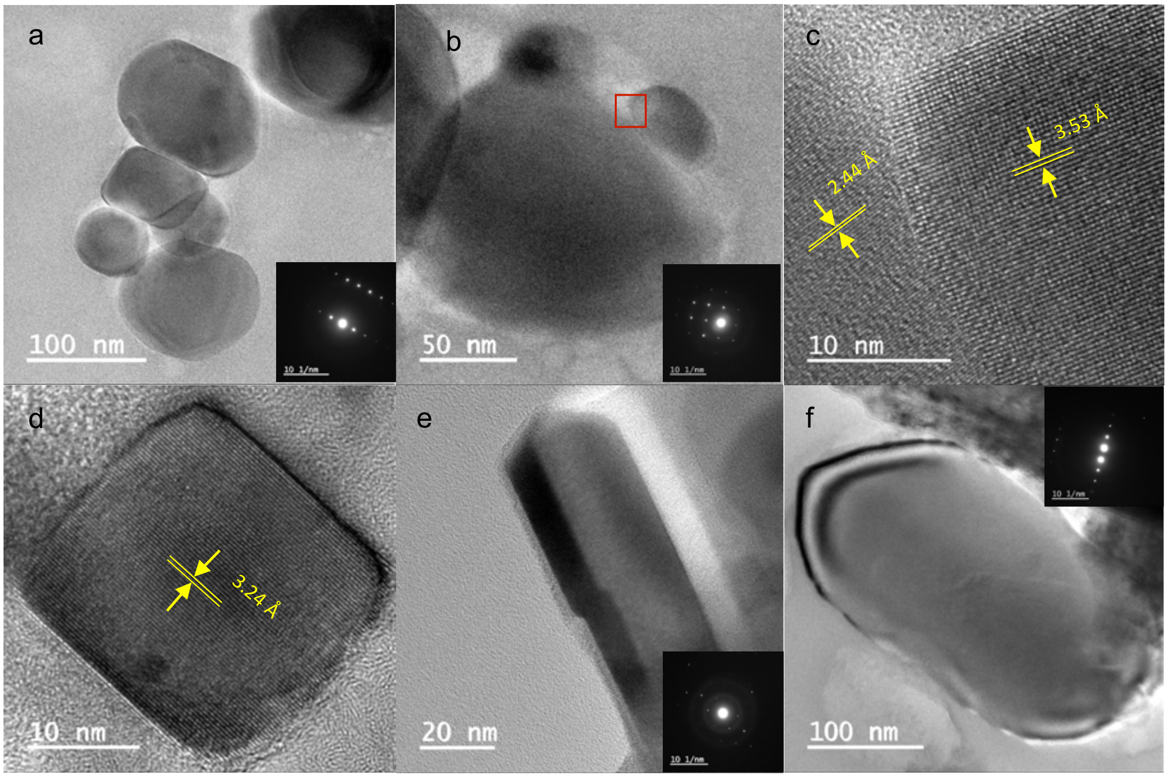

Supplementary Figure 1. Anatase and rutile observed in the Dan River sediment and coal ash samples: (**a**-**c**) anatase TiO_2 found in downstream river sediments (**c** is the magnification of the area denoted in **b**); (**d**-**e**) rutile particles found in the Dan River coal ash; (**f**) rutile particle found in the upstream Dan River sediment.

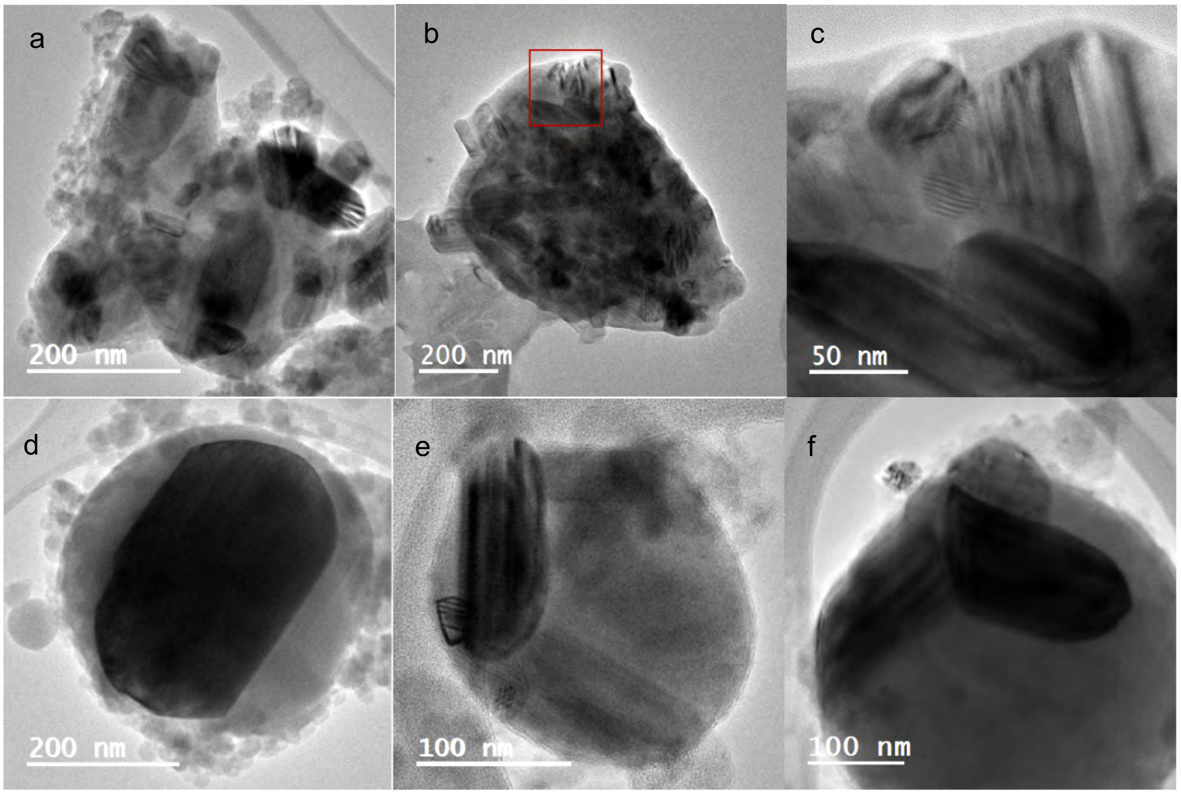

Supplementary Figure 2. The Magnéli phase of Ti oxides in various coal ashes, showing distinctive superstructure, typically characterized by striations. (See Table 1 for sample names and origins.) (**a**) Plant H fly ash 2015 with high-S feed coal; (**b**-**c**) Dan River coal ash (**c** is the magnification of the selected area in **b**); (**d**) China CZDC 2 flyash; (**e**) Plant H fly ash 2007 with low-S feed coal; (**f**) Plant I fly ash 2007 with low-S feed coal.

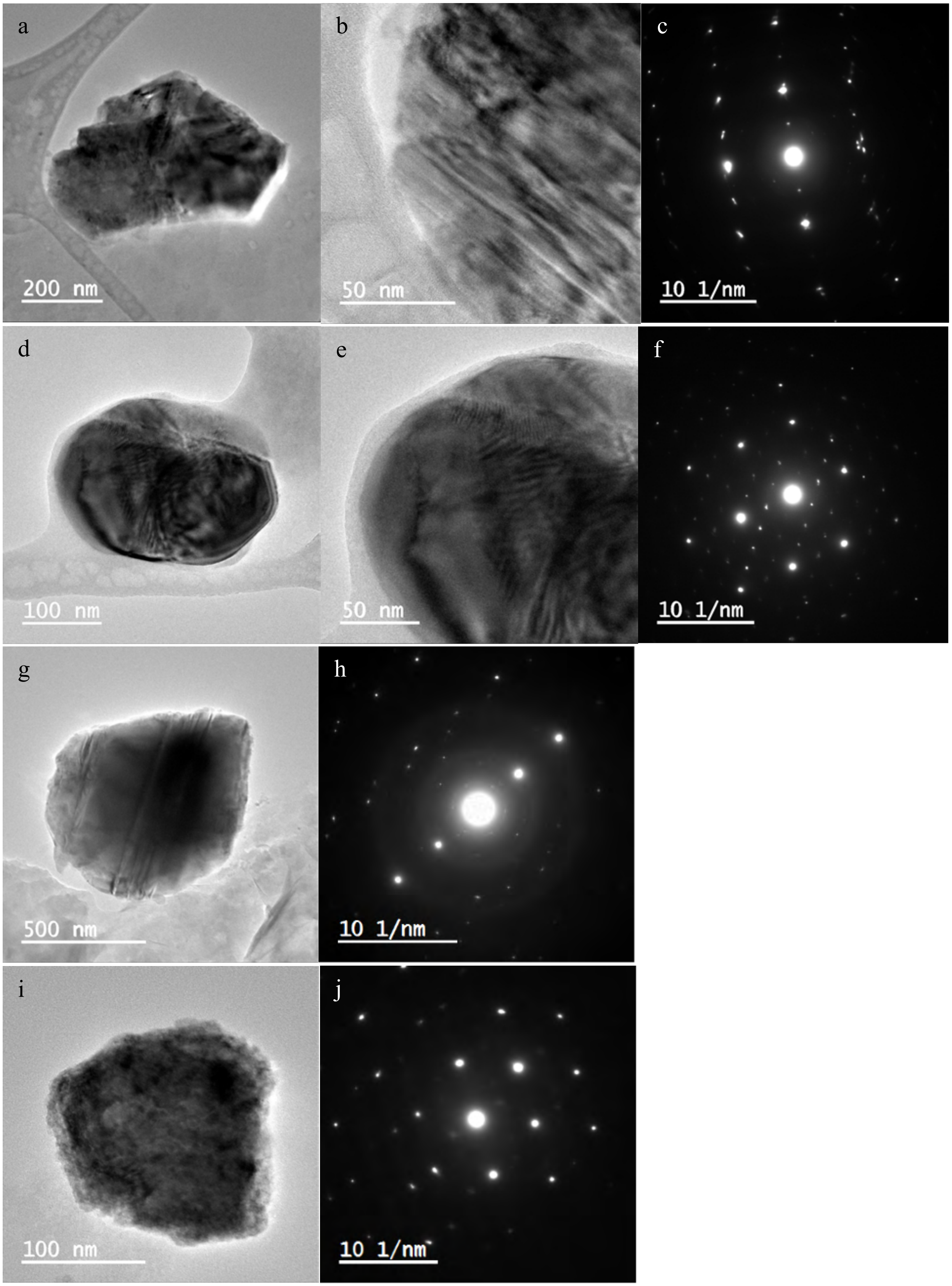

a
200 nm
b
50 nm
c
10 1/nm
d
100 nm
e
50 nm
f
10 1/nm
g
500 nm
h
10 1/nm
i
100 nm
j
10 1/nm

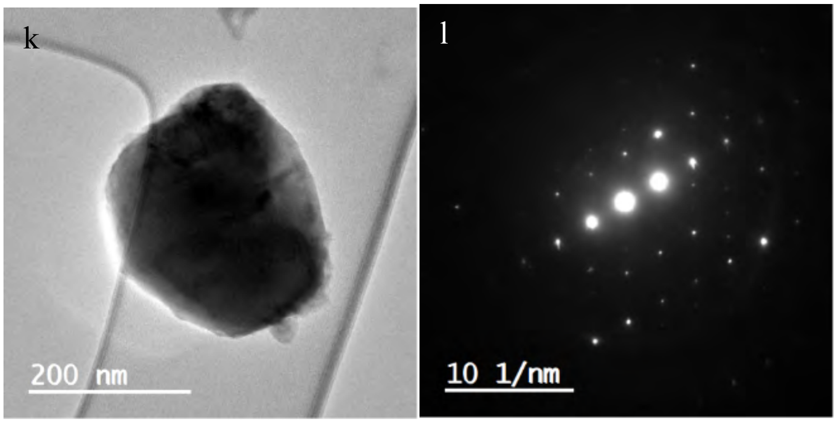

Supplementary Figure 3. Magnéli phases in a storm water pond sediment located in Durham, North Carolina, USA (**a**-**c**); in a dust sample taken on the road of Shanghai, China (**d**-**f**); in a small lake in North Carolina, USA, receiving runoff from a coal ash impoundment (**g**-**h**); in an estuarine sediment sample in China (**i**-**j**); and in a WWTP sludge in Shanghai, China (**k**-**l**).

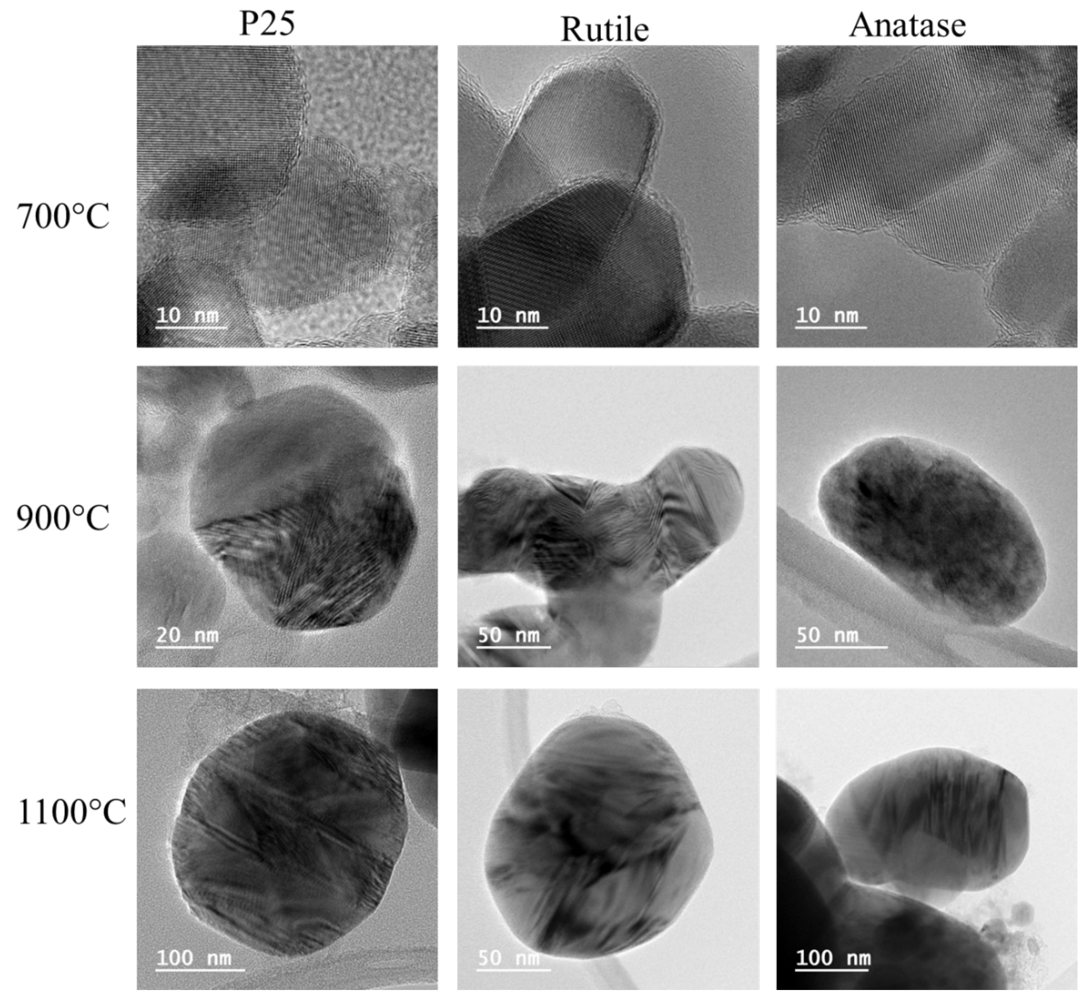

Supplementary Figure 4. TEM images of coal-annealed P25, rutile, and anatase nanoparticles at different temperatures. Conversion to Magnéli phases is apparent at 900°C and above. Although the characteristic nano-fine linear striations are apparent in all the Magnéli phases shown here, some of the images at 900°C and 1100°C show contrast waviness due to Moiré interference.

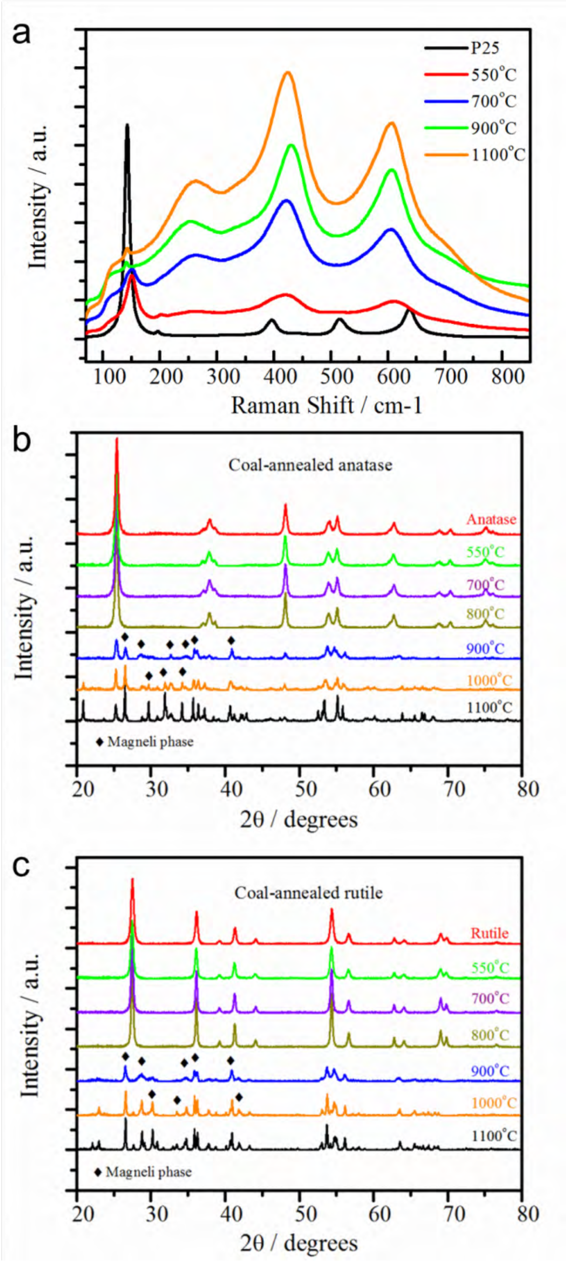

Supplementary Figure 5. (a) Raman spectra of the coal-annealed P25 TiO_2 powder at different temperatures. XRD patterns of coal-annealed titanium oxides at different temperatures with different initial TiO_2 powders: (b) anatase, and (c) rutile.

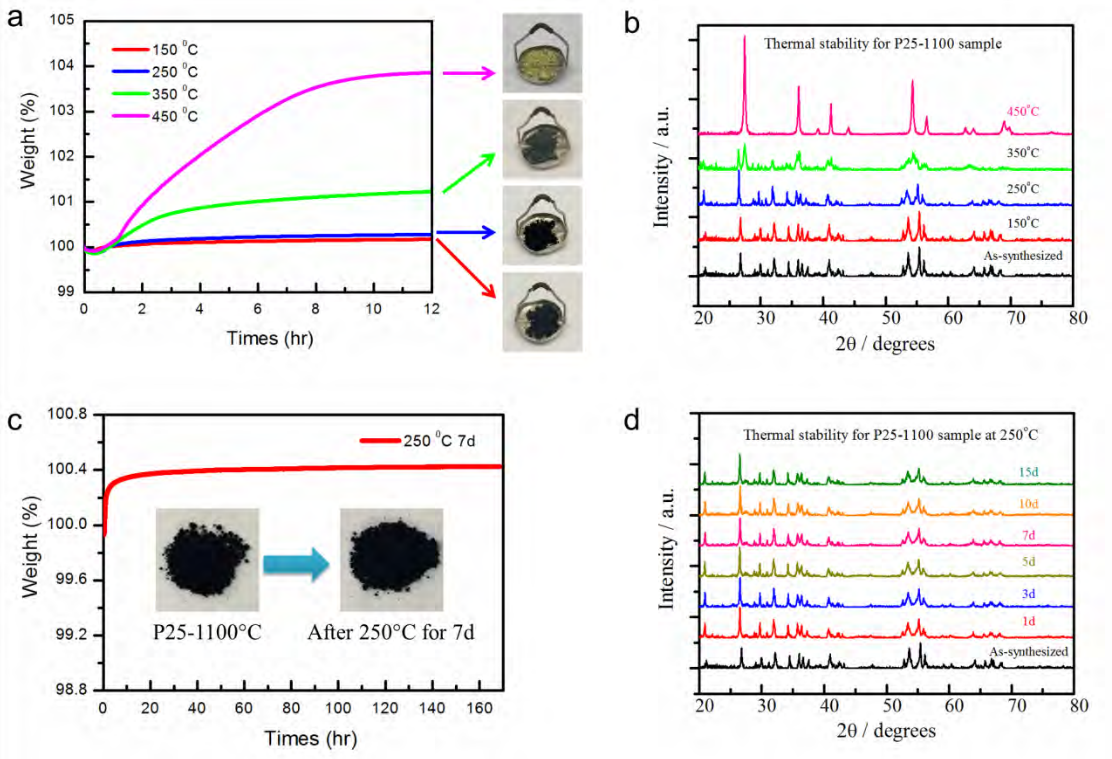

Supplementary Figure 6. (a) Stability analysis of Magnéli P25-1100°C sample by TGA at different temperatures for 12 hours and corresponding photographs of P25-1100°C after TGA measurement; (b) XRD patterns after TGA measurement; (c) TGA analysis of Magnéli P25-1100°C sample at 250°C for 7 days; (d) XRD patterns of Magnéli P25-1100°C sample after annealing at 250 °C for different times.

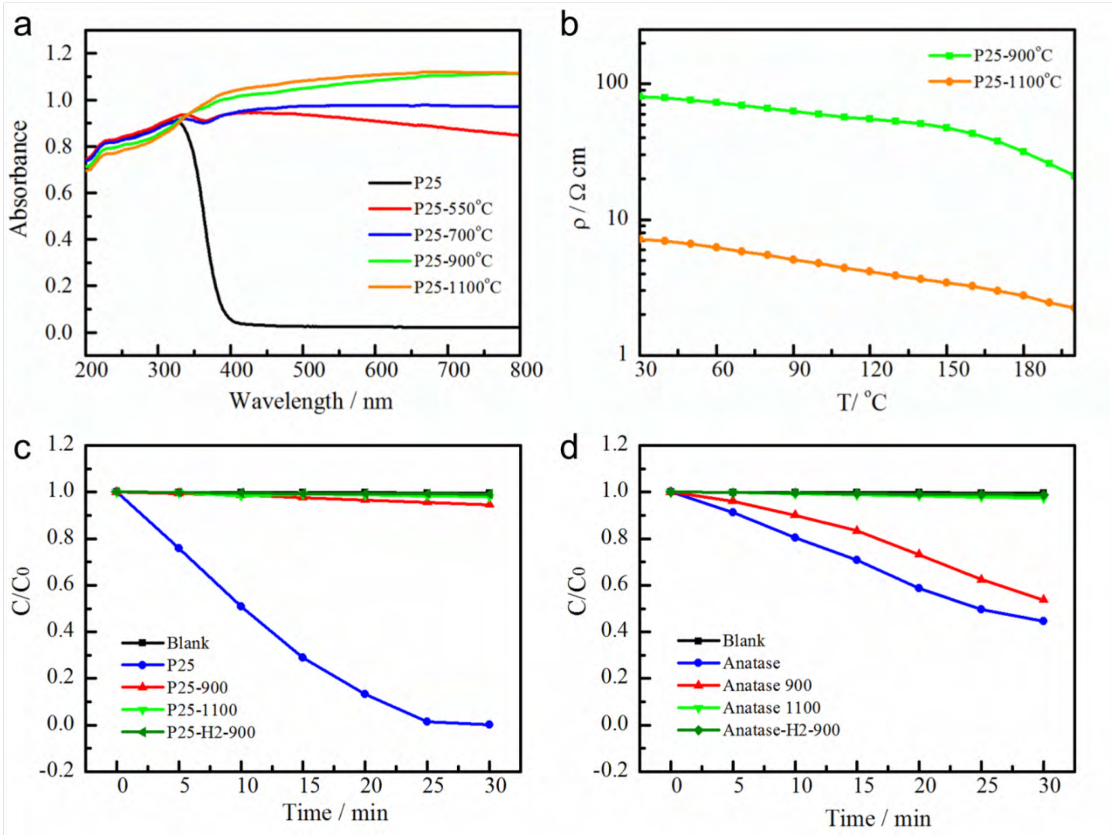

Supplementary Figure 7. (a) UV-vis absorption spectra for coal-annealed P25 TiO_2 powder at different temperatures. (b) Resistance relation with temperature for coal-annealed P25 samples. Photocatalytic performance (as measured by the degradation of methylene blue concentrations on the y-axis) of pristine and coal-annealed (c) P25 and (d) anatase titanium oxides under simulated full range sunlight.

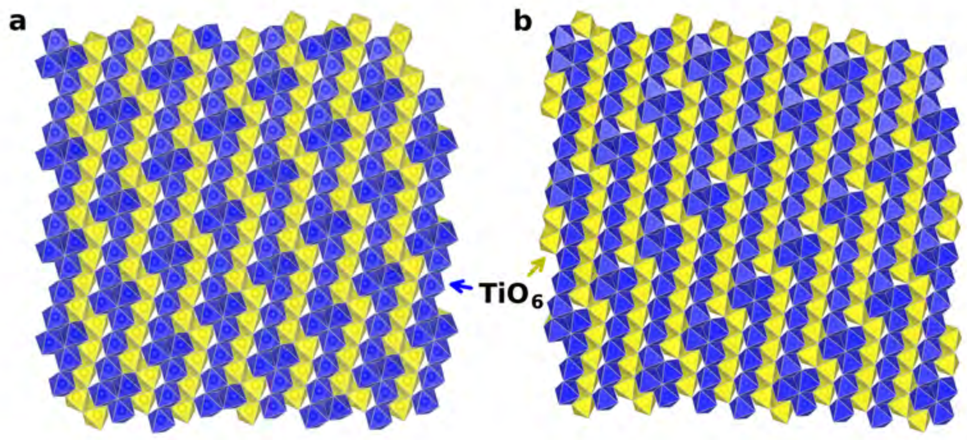

Supplementary Figure 8. Schematic illustration of two crystalline structures of Magnéli phases Ti_xO_{2x-1}: (a) Ti_5O_9 and (b) Ti_7O_{13}. Both structures are composed of chains of edge-sharing TiO_6 octahedra interrupted every x^{th} octahedron. The top and bottom layers are depicted in blue and yellow, respectively.

Supplementary Table 1. Phases of the Ti_xO_{2x-1} family (including rutile), their lattice cell constants (setting after Le Page and Strobel, 1982, space group symmetry -I), and their largest observed d-spacing (002) and corresponding two-theta value.

Phase	Unit Cell	Largest D spacing [Å] (002)[1]	Smallest value for two-theta[°] (Cu Kα)
Rutile	a = 5.46 Å<br>b = 7.14 Å<br>c = 2.96 Å<br>α= 66°<br>β* = 38°<br>γ = 108°	3.24	27.46
Ti_4O_7	c = 20.72 Å	6.2	14.37
Ti_5O_9	c = 26.63 Å	7.8	11.32
Ti_6O_{11}	c = 32.23 Å	9.5	9.27
Ti_7O_{13}	c = 38.15 Å	11.2	7.87
Ti_8O_{15}	c = 44.06 Å	12.9	6.83
Ti_9O_{17}	c = 50.03 Å	14.6	6.04

[1]SAED patterns commonly display deviations from these average values by ± 0.3 Å most likely due to additional disordering, shearing or impurities. In the case of Ti_6O_{11}, the most common Magnéli phase observed in fly ash and sediments, the d-spacing for (002) varied over a larger range (8.9 - 10.6 Å) suggesting the presence of phases intermediate in composition between Ti_5O_9 , Ti_6O_{11} and Ti_7O_{13}, respectively.

Supplementary Table 2. Environmental sample information

No.	Sample	Sample description
1	sediment	storm water pond in Durham, North Carolina, USA
2	sediment	Sutton Lake, North Carolina, USA, formally received effluent from coal ash ponds
3	sediment	Yangtze River Estuarine area in China
4	sludge	WWTP in Qinpu district in Shanghai, China
5	dust	street dust in an industrial area of Shanghai, China

Supplementary Table 3. Experimental observations of P25 annealed at 900ºC and 1100ºC in the presence of powdered coal under an N_2 atmosphere.

P25	not heated	rutile + anatase
P25	heated to 900ºC	Major<br>Ti_6O_{11}<br>Minor<br>Ti_5O_9<br>Ti_7O_{13}<br>Ti_9O_{17}<br>Other phases:<br>rutile<br>anatase<br>brookite
P25	heated to 1100 ºC	Major<br>Ti_4O_7<br>Ti_5O_9<br>Minor<br>Ti_6O_{11}<br>Ti_9O_{17}<br>Other phases:<br>rutile<br>anatase
